# Sympathovagal Balance Is a Strong Predictor of Post High-Volume Endurance Exercise Cardiac Arrhythmia

**DOI:** 10.3389/fphys.2022.848174

**Published:** 2022-03-21

**Authors:** Daniel W. T. Wundersitz, Bradley J. Wright, Brett A. Gordon, Stephanie Pompei, Carl J. Lavie, Voltaire Nadurata, Kimberly Nolan, Michael I. C. Kingsley

**Affiliations:** ^1^Holsworth Research Initiative, La Trobe Rural Health School, La Trobe University, Bendigo, VIC, Australia; ^2^School of Psychology and Public Health, La Trobe University, Melbourne, VIC, Australia; ^3^John Ochsner Heart and Vascular Institute, Ochsner Clinical School-The University of Queensland School of Medicine, New Orleans, LA, United States; ^4^Cardiology Department, Bendigo Health, Bendigo, VIC, Australia; ^5^Department of Exercise Sciences, University of Auckland, Auckland, New Zealand

**Keywords:** sympathovagal balance, autonomic imbalance, cardiac, arrhythmia, endurance

## Abstract

Regular physical activity is important for cardiovascular health. However, high-volume endurance exercise has been associated with increased number of electrocardiogram (ECG) abnormalities, including disturbances in cardiac rhythm (arrhythmias) and abnormalities in ECG pattern. The aim of this study was to assess if heart rate variability (HRV) is associated with ECG abnormalities. Fifteen participants with previous cycling experience completed a 21-day high-volume endurance exercise cycle over 3,515 km. Participants wore a 5-lead Holter monitor for 24 h pre- and post-exercise, which was used to quantify ECG abnormalities and export sinus R-to-R intervals (NN) used to calculate HRV characteristics. As noise is prevalent in 24-h HRV recordings, both 24-h and heart rate collected during stable periods of time (i.e., deep sleep) were examined. Participants experienced significantly more arrhythmias post high-volume endurance exercise (median = 35) compared to pre (median = 12; *p* = 0.041). All 24-h and deep sleep HRV outcomes were not different pre-to-post high-volume endurance exercise (*p* > 0.05). Strong and significant associations with arrhythmia number post-exercise were found for total arrhythmia (total arrhythmia number pre-exercise, ρ = 0.79; age, ρ = 0.73), supraventricular arrhythmia (supraventricular arrhythmia number pre-exercise: ρ = 0.74; age: ρ = 0.66), and ventricular arrhythmia (age: ρ = 0.54). As a result, age and arrhythmia number pre-exercise were controlled for in hierarchical regression, which revealed that only deep sleep derived low frequency to high frequency (LF/HF) ratio post high-volume endurance exercise predicted post total arrhythmia number (*B* = *0.63, R*^2^Δ = 34%, *p* = 0.013) and supraventricular arrhythmia number (*B* = *0.77, R*^2^Δ = 69%, *p* < 0.001). In this study of recreationally active people, only deep sleep derived LF/HF ratio was associated with more total and supraventricular arrhythmias after high-volume endurance exercise. This finding suggests that measurement of sympathovagal balance during deep sleep might be useful to monitor arrhythmia risk after prolonged high-volume endurance exercise performance.

## Introduction

Regular aerobic exercise is important for cardiovascular (CV) health (Hower et al., 2018) and decreases the risk of CVdiseases (CVD), such as myocardial infarction and heart failure (Channon, 2020). It also leads to positive chronic structural and functional adaptations to the heart (Sharma, 2003) which are more apparent if the recommended volume of exercise per week is exceeded (George et al., 2012). This has led to an increasing proportion of the general population performing aerobic exercise on a weekly basis for periods of time that greatly exceed exercise recommendations for health (Piercy et al., 2018) (termed high-volume endurance exercise). However, links between high-volume endurance exercise and adverse CV changes are increasingly being reported (Williams and Thompson, 2014; Elliott and La Gerche, 2015), such as sudden cardiac death (Tsuji et al., 1996) and electrocardiogram (ECG) abnormalities (Ector et al., 2007; Abdulla and Nielsen, 2009; Wundersitz et al., 2019). ECG abnormalities include disturbances in the cardiac rhythm (arrhythmias) and abnormalities in the ECG pattern. Arrhythmias can be further classified as those originating in the ventricles (ventricular arrhythmias) or in the atria and other regions of the heart above the ventricles (supraventricular arrhythmias). For example, a 10-fold increase in cardiac arrhythmias was found in adults pre-to-post high-volume endurance exercise performance previously. High-volume endurance exercise is also being performed by people with varied training histories and across the lifespan, which may also be of concern for older adults performing this form of exercise as age is related to ECG abnormality (Zorzi et al., 2018b; Mannina et al., 2021). An ability to understand when the heart is acutely under excess stress from the chronic performance of high-volume endurance exercise would be beneficial for safe high-volume endurance exercise performance for those in the general population, as well as reducing heart injury risks that can be severe and potentially life threatening.

Imbalances in autonomic control are increasingly being linked to chronic ECG abnormality in the literature (Task Force of the European Society of Cardiology and the North American Society of Pacing and Electrophysiology, 1996; Sareban et al., 2020), especially in clinical populations (e.g., Smith, 1982; Binkley et al., 1991; Bigger et al., 1992; Algra et al., 1993; Kocovic et al., 1993; Hartikainen et al., 1996; de Oliveira et al., 2014). A relatively simple and non-invasive method that has shown promise for monitoring autonomic control is through heart rate variability (HRV) assessment, which is used to measure changes in intervals between successive heartbeats and is a non-invasive reflection of the health of the cardiac automatic nervous system (ANS) (Rajendra Acharya et al., 2006). A healthy ANS normally displays variability in heart rate and a stressed system is usually less variable (Kleiger et al., 1987; Nolan et al., 1998; La Rovere et al., 2003). Although, elevations in atrial fibrillation (AF) is strongly associated with increased risk of mortality (Bigger et al., 1992) and arrhythmias like AF can lead to elevations in HRV (e.g., low frequency to high frequency [LF/HF] ratio Lombardi et al., 2001).

It has been previously demonstrated that high-volume endurance exercise changes HRV in athletes (Zehender et al., 1990; Carter et al., 2003; Hynynen et al., 2006; Swanson, 2006; Seiler et al., 2007; Seidl and Asplund, 2014). For example, Seiler et al., 2007 found that highly trained athletes displayed changes in HRV, lasting 90 min after performing 60 to 120 min of exercise, and Hynynen et al. (2006) found chronic overtraining in athletes lead to significantly decreased HRV characteristics. Although, HRV analysis appears to provide a useful evaluation of autonomic control in athletes, reflecting the hearts ability to recover and respond to changing exercise-related stimuli (Acharya et al., 2007) a potential limitation of this method is that any activity during waking hours, such as physical movement (Moody, 1992; Saponznikov et al., 1992; Tobaldini et al., 2013) can introduce errors or noise within the signal. Hence, potentially important changes in ANS control might be missed. A more reliable method of assessing HRV, is to section and analyze data for periods where participants heart rate is stable, such as during deep sleep (Clifford et al., 2005; Herzig et al., 2017a,b; Kim and Shin, 2017). Deep sleep refers to sleep periods where there is regular breathing, high delta wave activity and low variation in HRV characteristics (Herzig et al., 2017b). Previous authors have reported that deep sleep HRV is more time accurate and reliable when compared to waking hours and other restful periods (Catcheside et al., 2001).

Deep sleep HRV has been assessed in several studies of athletes, but only shortly after an acute bout of exercise (Al Haddad et al., 2009; Myllymaki et al., 2012; Herzig et al., 2018). For example, Al Haddad et al., 2009 assessed the effect of supramaximal intermittent exercise to exhaustion on deep sleep HRV and found post-exercise vagal-related HRV characteristics significantly changed immediately and 12 h after exercise (i.e., LF/HF ratio expressed as a percentage significantly higher, mean RR significantly lower), but had returned to normal after 36 h. To date, however, no study has assessed the effect of high-volume endurance exercise on deep sleep HRV or investigated if changes in HRV are related to ECG abnormalities in response to high-volume endurance exercise. Therefore, the aim of this study was to assess the effect of high-volume endurance exercise in recreationally active people on HRV characteristics and if changes in HRV characteristics are related to ECG abnormalities in response to high-volume endurance exercise.

## Materials and Methods

### Participants and Protocol

This study performed a secondary analysis on previously published data (Wundersitz et al., 2019). The study was approved by the Bendigo Health Care Group Human Research Ethics Committee and the La Trobe University Human Ethics Committee (HREC/17/BHCG/9). Participants or legal guardians of participants aged under 18 years provided written informed consent prior to participating. Fifteen (14 male) recreationally active people (mean ± SD: age, 34.7 ± 19.3 years; height, 1.79 ± 0.08 m; body mass, 78.3 ± 17.6 kg) with 8.4 ± 12.8 years of recreational cycling experience took part in the ‘Make a difference, change our world’ charity bicycle ride (MADRIDE)^1^. The ride covered 3,515 km from Bunbury in Western Australia to Bendigo in Victoria (similar in distance to the Tour De France) (Marijon et al., 2013). Prior to completing the ride, participants completed screening (Balady et al., 1998) and training history questionnaires. Participants reported training for 8.7 (95%CI 4.9 – 12.5) months and covering 3.6 (3.1 – 4.2) sessions per week. On average during each session they trained for 2.9 (2.1 – 3.6) hours and covered 78 (63.5 – 92.5) km. Participants cycled for 21 consecutive days, rode for 6.4 (5.8 – 6.9) hours a day and on average covered 167 (150.9 – 183.8) km/day at 26.3 (25.2 – 27.4) km/h.

To determine HRV characteristics and ECG abnormality number, a 5-lead Holter monitor (SEER Light; GE Healthcare, Horten, Norway) was placed on each participants chest by a cardiac technician and worn for 24 h (9.2 ± 3.2 days pre-exercise). Whilst wearing the Holter monitor, participants were instructed to avoid exercising and to limit intense activities of daily living during this time. Participants again wore the same Holter monitor after completing the 21-days of high-volume endurance exercise (3.0 ± 1.8 days post-exercise). The availability of Holter monitors for research in a public hospital setting limited the ability to test all participants immediately post multi-day high-volume endurance exercise and was added as a covariate to analyses. Holter monitoring was chosen as it is the gold standard method of HRV measurement (Akintola et al., 2016; Georgiou et al., 2018).

### Data Analysis

Manufacturer software (version 8, MARS Holter Analysis System; GE Healthcare, Horten, Norway) was used to process raw Holter monitor data throughout both 24-h wear periods. Holter data were screened to identify ECG abnormalities by an experienced cardiac technician and verified by a cardiologist who were both blinded to participant identity and measurement time point. R-to-R intervals between QRS complexes (RR intervals) were then exported using QRSDK software (MARS Holter Analysis System version 8; GE Healthcare, Horten, Norway) to allow further analyses. Abnormal RR intervals (non-sinus rhythm) were excluded from the analysis and those normal intervals between QRS complexes not excluded (NN intervals) were saved for further analyses in LabView software (version 2016; National Instruments, United Kingdom). Specifically, the NN signal was visually displayed in LabView and then deep sleep periods were identified and exported. To be classified as a deep sleep period, a 10-min duration where NN intervals were stationary was required and this inactive period was considered to be when the NN interval had less than a 1% change in slope (Brandenberger et al., 2005). The middle 5-min within this 10-min window was then used to calculate time and frequency domain HRV outcomes. Specifically, time domain variables included: heart rate (HR) mean, NN mean, standard deviation of the NN intervals (NNSD), proportion derived by dividing NN50 by the total number of NN intervals (PNN50) and square root of the mean squared difference of successive NN intervals (RMMSD). Frequency domain variables included: Total power (the sum of the four spectral densities; low-frequency component [LF], high-frequency component [HF], ultra-low frequency [ULF] and very-low frequency component [VLF]), normalized power in the low frequency (LF norm), normalized power in the high frequency (HF norm) and LF/HF ratio. Time and frequency domain variables were analyzed according to recommendations by Malik et al. (1996) In addition, Poincaré SD1 (length of the transverse line of the Poincaré plot area) was calculated to quantify changes in parasympathetic nervous system activity and Poincaré SD2 (length of the longitudinal line of the Poincaré plot area) was calculated to quantify changes in sympathetic nervous system activity (Brennan et al., 2001). Then the ratio of the two (SD1/SD2) was calculated to represent the balance between parasympathetic and sympathetic nervous system activity. Poincaré analyses can be used to detect irregularities that may otherwise be difficult to determine with conventional time and frequency domain variables (Laitio et al., 2000).

### Statistical Analyses

SPSS for Windows (version 25; IBM Corporation, Armonk, NY, United States) was used to perform all statistical analyses. Shapiro-Wilks tests were performed to assess normality of data. If data were not-normally distributed, the data were log transformed. To assess the effect of 21-days of high-volume endurance exercise on HRV, one-way analysis of variance (ANOVAs - within factor: measurement time) were performed. Mauchly’s test was consulted for all ANOVA’s and Greenhouse Geisser corrected *p*-values are presented when the assumption of sphericity was violated. If significant main effects were found, pairwise comparisons with Bonferroni correction were performed. To explore the effect ‘participant age’ and the ‘measurement time point post cycling’ that data were captured, both were added as a covariates and additional ANCOVAs performed if significant Pearson’s correlation coefficients were identified between the covariate and dependent variable (Kim, 2018). Data were conveyed as the mean and 95% confidence interval unless data were not normally distributed (then the data were presented as median and interquartile range). Effect sizes (partial eta-squared statistic; η^2^_p_) are presented and statistical significance was set as *p* < 0.05.

To identify if there were factors that might predict post high-volume endurance exercise arrhythmia response, associations between participant characteristics (i.e., age, pre-exercise arrhythmia, training history, weekly training distance, measurement time point, gender) and all 24 h and deep sleep HRV pre/post characteristics were assessed with Spearman Rho correlations (ρ). Next, a two-step hierarchical regression was performed to identify which HRV characteristics were most associated with arrhythmia response (total arrhythmia, as well as supraventricular and ventricular arrhythmia responses) post multi-day endurance exercise. For the arrhythmia data, three (1 pre, 2 post) scores were 1.96 standard deviations above the mean and positively skewed the variables. Given that outlier adjustment is preferable to whole variable transformation in samples with 1 or 2 outliers, we winsorized (Rivest, 1994) the outliers whereby scores were adjusted down, while maintaining their rank. The pre and post multi-day endurance exercise arrhythmia data were normally distributed after winsorizing.

## Results

ECG abnormalities that were not classified as arrhythmias were noted in seven participants, three pre (2^nd^ degree AV block [two] and ST wave elevation) and four post (1^st^ degree AV block, 2^nd^ degree AV block, and ST-T wave changes [two]). In addition, single beat abnormalities were noted in three participants (Pre: bundle branch block, non-conducted atrial ectopic and ventricular escape beat; Post: junctional escape beat). Participants experienced significantly less total arrhythmias pre high-volume endurance exercise (median = 12, interquartile range = 4 – 62) compared to post (median = 35, interquartile range = 4 – 231; F_1_, _14_ = 5.038, p = 0.041, η^2^_p_ = 0.265). However, the differences pre to post high-volume endurance exercise in supraventricular (pre: median = 37, interquartile range = 3 – 40; post: median = 53, interquartile range = 4 – 57; F_1_, _14_ = 0.903, p = 0.358, η^2^_p_ = 0.061) and ventricular (pre: median = 6, interquartile range = 0 – 6; Post: median = 20, interquartile range = 0 – 20; F_1_, _14_ = 1.060, p = 0.321, η^2^_p_ = 0.07) arrhythmias did not reach significance.

### 24 h and Deep Sleep HRV Pre and Post High-Volume Endurance Exercise

There was an average of 5.8 (95%CI: 4.7 to 6.9) deep sleep periods per person identified at pre and 5.9 (95%CI: 4.8 to 6.9) identified post high-volume endurance exercise (F_1_, _14_ = 0.006, p = 0.938, η^2^_p_ < 0.001). 24-h and deep sleep HRV characteristics pre and post high-volume exercise are shown in Tables 1, 2. There was no statistically significant effect of measurement time on 24-h (*p* > 0.05, η^2^_p_ ≤ 0.139) or deep sleep (*p* > 0.05, η^2^_p_ ≤ 0.110) HRV characteristics measured.

**TABLE 1:** Time domain HRV characteristics calculated pre and post high-volume endurance exercise (*n* = 15).

HRV variable	Pre (95%CI)	Post (95%CI)	Mean difference (95%CI)	Effect size (η^2^)	*P* value
***24-h HRV***					
pNN50 (%)	28.0 (20.1 – 36.0)	27.7 (19.5 – 36.0)	−0.30 (−3.83 – 3.23)	0.002	0.857
RMSSD (ms) ^#^	63.9 (48.9 – 79.0)	64.0 (49.9 – 78.0)	0.06 (−5.08 – 5.20)	0.001	0.927
HR mean (bpm)	70.6 (66.5 – 74.7)	68.3 (63.3 – 73.3)	−2.31 (−5.26 – 0.63)	0.168	0.114
NNSD (ms)	73.6 (62.3 – 84.9)	74.3 (63.3 – 85.3)	0.69 (−3.49 – 4.87)	0.009	0.937
NN mean (ms)	906 (853 – 958)	932 (868 – 997)	26.9 (−11.5 – 65.3)	0.139	0.156
***Deep Sleep HRV***					
pNN50 (%)	45.0 (33.3 − 56.7)	40.5 (29.9 − 51.1)	−4.5 (−12.4 – 3.4)	0.097	0.240
RMSSD (ms)	79.8 (61.3 − 98.3)	73.1 (57.8 − 88.4)	−6.7 (−18.8 – 5.4)	0.092	0.254
HR mean (bpm)	55.2 (51.6 − 58.9)	56.0 (52.6 − 59.4)	0.8 (−2.2 – 3.8)	0.021	0.589
NNSD (ms)	79.5 (64.1 – 95.0)	78.2 (64.9 – 91.4)	−1.4 (−11.6 – 8.9)	0.006	0.781
NN mean (ms)	1116 (1038 − 1195)	1099 (1037 − 1161)	−17.3 (−80.9 – 46.3)	0.024	0.569

*Data are presented as mean (95%CI). Note: ^#^, data were not normally distributed; η^2^_p_, partial eta-squared statistic; bpm, beats per minute; HR, heart rate; ms, millisecond; NN mean, mean of normal-to-normal intervals between QRS complexes; NNSD, standard deviation of the NN intervals; pNN50, proportion derived by dividing NN50 by the total number of NN intervals; RMSSD, the square root of the mean of the sum of the squares of differences between adjacent normal R-R intervals SD, standard deviation; SDNN, SD of all NN intervals.*

**TABLE 2:** Frequency domain HRV characteristics calculated pre and post high-volume endurance exercise (*n* = 15).

HRV variable	Pre (95%CI)	Post (95%CI)	Mean difference (95%CI)	Effect size (η^2^)	*P* value
***24-hour HRV***					
Total power (ms^2^) ^#^	5516 (3896 – 7136)	5540 (4049 – 7030)	23.5 (−590 – 638)	0.011	0.697
LF norm	67.9 (62.1 – 73.6)	67.6 (62.1 – 73.1)	−0.24 (−3.25 – 2.76)	0.002	0.865
HF norm	28.7 (23.2 – 34.1)	29.1 (23.9 – 34.3)	0.45 (−2.17 –3.08)	0.010	0.717
LF/HF ratio	2.77 (2.03 – 3.50)	2.68 (1.99 – 3.37)	−0.09 (−0.41 – 0.24)	0.015	0.646
Poincaré SD1 (ms) ^#^	45.3 (34.6 – 55.9)	45.3 (35.4 – 55.2)	0.04 (−3.59 – 3.67)	0.001	0.926
Poincaré SD2 (ms)	272 (237 – 307)	254 (225 – 282)	−18.4 (−47.6 – 10.9)	0.115	0.199
SD1/SD2 ratio	0.16 (0.14 – 0.19)	0.17 (0.15 – 0.20)	0.01 (−0.01 – 0.03)	0.102	0.228
***Deep sleep HRV***					
Total power (ms^2^)	6145 (4040 - 8250)	6590 (4243 - 8936)	444 (−991 – 1880)	0.031	0.518
LF norm	55.2 (43.6 - 66.7)	58.8 (49.2 - 68.5)	3.67 (−5.9 – 13.2)	0.045	0.423
HF norm	41.3 (30.3 - 52.2)	37.9 (28.7 - 47.1)	−3.4 (−11.8 – 5.1)	0.050	0.406
LF/HF^#^	1.40 (0.71 – 1.65)	1.36 (1.16 – 1.66)	0.10 (−0.20 – 0.40)	0.062	0.352
Poincaré SD1 (ms)	56.6 (43.5 - 69.7)	51.9 (41.1 - 62.7)	−4.7 (−13.3 – 3.8)	0.091	0.256
Poincaré SD2 (ms)	112.7 (93.8 - 131.6)	120.2 (100.1 - 140.4)	7.5 (−12.0 – 27.0)	0.047	0.422
SD1/SD2 ratio ^#^	0.72 (0.65 – 0.77)	0.68 (0.61 – 0.77)	−0.04 (−0.11 – 0.03)	0.110	0.210

*Data are presented as mean (95%CI). ^#^, data were not normally distributed; η^2^_p_, partial eta-squared statistic; HF norm, normalized power in the high frequency; LF norm, normalized power in the low frequency; LF/HF, ratio of LF to HF power; Poincaré SD1, length of the transverse line of the Poincaré plot area; Poincaré SD2, length of the longitudinal line of the Poincaré plot area; SD1/SD2 ratio, Poincaré SD1 to Poincaré SD2 ratio.*

## Factors Associated With Arrhythmia Response Post High-Volume Endurance Exercise

Age was the only factor to strongly correlate with total, supraventricular and ventricular arrhythmia post high-volume endurance exercise (ρ ≥ 0.54, *p* < 0.05). Arrhythmia pre strongly correlated with total and supraventricular arrhythmia post high-volume endurance exercise (ρ ≥ 0.66, *p* < 0.01), but not ventricular arrhythmia (ρ = 0.27, *p* < 0.05). All other factors were not significant (*p* > 0.05) and displayed moderate or lower correlations (ρ ≤ 0.43) with arrhythmia response post-exercise (Table 3). Deep sleep and 24-h HRV correlations are provided in Supplementary Data.

**TABLE 3:** Correlations comparing arrhythmia, age, days post-ride measurement performed, riding years and weekly training distance.

	Arrhythmia	1	2	3	4	5	6	7
(1). Arrhythmia Post^WIN^	Total	1						
	Supraventricular	1						
	Ventricular	1						
(2). Arrhythmia Pre^WIN^	Total	0.79**	1					
	Supraventricular	0.66**	1					
	Ventricular	0.27	1					
(3). Age	Total	0.73**	0.67**	1				
	Supraventricular	0.74**	0.69**	1				
	Ventricular	0.54*	0.18	1				
(4). Measurement time	Total	−0.19	−0.13	−0.39	1			
	Supraventricular	−0.14	−0.09	−0.39	1			
	Ventricular	−0.23	−0.01	−0.39	1			
(5). Riding years	Total	−0.26	−0.11	0.01	−0.21	1		
	Supraventricular	−0.33	−0.09	0.01	−0.21	1		
	Ventricular	0.00	−0.27	0.01	−0.21	1		
(6). Distance per week	Total	−0.19	−0.06	−0.37	−0.15	0.15	1	
	Supraventricular	−0.15	−0.06	−0.37	−0.15	0.15	1	
	Ventricular	−0.05	0.07	−0.37	−0.15	0.15	1	
(7). Gender	Total	0.43	0.37	0.31	−0.27	0.03	−0.09	1
	Supraventricular	−0.06	0.06	0.31	−0.27	0.03	−0.09	1
	Ventricular	0.44	0.49	0.31	−0.27	0.03	−0.09	1

* p < 0.05; ** p < 0.01; ^WIN^, outliers were winsorized whereby scores were adjusted down, while maintaining their rank.

### Does HRV Predict Post High-Volume Endurance Exercise ECG Predict Total Arrhythmia, Supraventricular and Ventricular Number

In the final hierarchal regression model that used the stepwise entry method at step 2, only deep sleep derived LF/HF ratio post high-volume endurance exercise was included in the final model for total arrhythmia and supraventricular arrhythmia analyses after controlling for age and pre-exercise arrhythmia number (Table 4). Specifically, higher LF/HF ratio post high-volume endurance exercise was strongly associated with higher counts of arrhythmia (total arrhythmia: B = 0.80, *p* = 0.012). The full model with all variables included was also a significant predictor of post-exercise total arrhythmia, R^2^ = 0.64, F(3, 10) = 6.01, *p* = 0.013 and supraventricular arrhythmia R^2^ = 0.69, F(3, 10) = 22.13, *p* < 0.001. Post exercise ventricular arrhythmia during deep sleep and the 24-h data (total arrhythmia, supraventricular and ventricular arrhythmia post exercise), were not associated with participant age or arrhythmia pre-exercise and no additional variables were entered at step 2.

**TABLE 4:** Hierarchical regression with a Stepwise solution after step 1 assessing if measures of 24-h and deep sleep HRV are associated with post exercise ECG abnormality and arrhythmia after controlling for age and pre-exercise abnormality (*n* = 14).

		Step summary					
		DS HRV			24-H HRV		
	Predictor	*Beta*	*R*^2^Δ	*p*	*Beta*	*R*^2^Δ	*p*
Total arrhythmia number	Step 1		0.30	0.136		0.18	0.334
	Age	−0.25			0.36		
	Arrhythmia pre-exercise	0.23			0.30		
	Step 2		0.34	0.013		–	–
	LF/HF post exercise	0.80*			–		
Supraventricular arrhythmia	Step 1		0.18	0.334		0.18	0.344
	Age	−0.33					
	Arrhythmia pre-exercise	0.25			0.35 0.31		
	Step 2		0.69	< 0.001		–	–
	LF/HF post exercise	1.07**			–		
Ventricular arrhythmia	Step 1		0.02	0.887		0.02	0.887
	Age	0.12			0.12		
	Arrhythmia pre-exercise	−0.10			–0.10		

* p < 0.05; ** p < 0.01; R^2^Δ = R^2^ change; DS HRV, heart rate variability derived during deep sleep; 24-H HRV, heart rate variability derived during 24-h recording.

### Additional Findings

We removed the one female participant from the regression analyses as controlling for gender with such an imbalance is not appropriate. However, to confirm if the trends observed would remain with the additional female participant, we re-ran the analysis and controlled for gender. In this model, the findings mirrored those from total arrhythmia, with higher LF/HF post-exercise only associated with all post-exercise ECG abnormality (*B* = *0.80, p* = 0.012) and supraventricular arrhythmia (*B* = 1.07, *p* < 0.001) during deep sleep.

## Discussion

This was the first study to assess the relationship between deep sleep HRV characteristics, using a controlled measurement technique, and number of ECG abnormality in a recreational population of cyclists after performing multi-day high-volume endurance exercise. The main finding was that during deep sleep LF/HF ratio was a strong predictor of post-exercise total and supraventricular arrhythmia after controlling for age and arrhythmia number pre-exercise. We also confirmed that baseline arrhythmia number, in particular total and supraventricular arrhythmia number, and age were strongly correlated with post-exercise arrhythmia response, and that HRV characteristics were not significantly changed after performing multi-day high-volume endurance exercise.

Results from the current study suggest that post multi-day high-volume endurance exercise total arrhythmia and supraventricular number were related to baseline arrhythmia number and participant age. This result supports the widespread understanding that age is related to ECG abnormality, both in athletes (Andersen et al., 2013; Zorzi et al., 2018a,b) and the general population (Furberg et al., 1994; Lok and Lau, 1996; Mannina et al., 2021). This result is likely influenced by cardiovascular system deterioration with increasing age (Hatch et al., 2011) and accumulated demand on the heart from cumulative years of exercise training (Myrstad et al., 2014). The association between baseline total arrhythmia number with post-exercise total arrhythmia response suggests that multi-day high-volume endurance exercise is not likely to influence ECG abnormality in people with baseline arrhythmias.

Supraventricular arrhythmia number post high-volume endurance exercise was also related to baseline supraventricular number. This result is not unexpected, considering that high volume endurance exercise is accepted as a factor contributing to the development of supraventricular arrhythmias (D’Souza et al., 2019), including bradyarrhythmia, atrioventricular block (Baldesberger et al., 2008) and atrial fibrillation (Andersen et al., 2013; Myrstad et al., 2014). Further, supraventricular arrhythmias can have serious quality of life and sport participation consequences in athletes (D’Souza et al., 2019), although in the general population it has been suggested that they are common and rarely life threatening (Blomstrom-Lundqvist et al., 2003). The current findings highlight the importance of screening programs being performed prior to high-volume endurance exercise to identify unknown and potentially serious cardiac issues (Corrado et al., 2006; Mont et al., 2020).

Controlling for age and baseline arrhythmia number, the current results showed that higher LF/HF ratio post-exercise, when recorded during deep sleep, was a strong predictor of post-exercise total arrhythmia and supraventricular arrhythmia number. This finding was not seen when LF/HF ratio data were analyzed over a 24-h period or when ventricular arrhythmias were assessed. To our knowledge, researchers have not previously assessed the effect of endurance exercise in humans on HRV measures (during deep sleep or 24 h) and used these to predict ECG abnormality response. There has been HRV and ECG abnormality research in one study of horses performing endurance exercise (Flethøj et al., 2016), as well as animal and clinical populations performing single (e.g., Guiraud et al., 2013; Broux et al., 2017; Frick et al., 2019) or multiple (training studies; e.g., Deligiannis et al., 1999; Andrade et al., 2017) sessions of short duration exercise. In these studies, mixed findings were found between LF/HF ratio and ECG abnormality. For example, similar to the current study Guiraud et al. (2013), found an association between ECG abnormality and LF/HF ratio, with a decrease in ECG abnormality strongly related to a decrease in LF/HF ratio in people with chronic heart failure performing high intensity exercise (*r* = 0.66; *p* < 0.01). However Broux et al. (2017), found that LF/HF ratio was significantly lower at rest and during exercise (lunging test) in horses with atrial fibrillation (*p* < 0.001). Although few exercise studies are available, there is an abundance of research in clinical populations demonstrating that either a higher (Lombardi et al., 2001; Rizzo et al., 2015) or lower (Nortamo et al., 2018; Sagnard et al., 2020) LF/HF ratio is associated with ECG abnormality. There are a number of potential factors alone or in combination that might contribute to inconsistent findings in the literature, such as the duration over which HRV was assessed (Furlan et al., 1990), as well as the population (i.e., animal or healthy/clinical human) and type of ECG abnormality (Lombardi et al., 2001) under investigation. For example, mechanisms responsible for heart rate control are not likely to, and cannot, be considered stable with longer term recordings, such as over 24-h (Furlan et al., 1990). Performing moderate physical activity (Moody, 1992; Saponznikov et al., 1992; Tobaldini et al., 2013), emotional circumstances/mental stress (Task Force of the European Society of Cardiology and the North American Society of Pacing and Electrophysiology, 1996), and changing respiration (Malliani et al., 1991) or blood pressure (i.e., hypotension) can all induce changes in LF or HF power, hence LF/HF ratio, across longer term recordings. These should be controlled for and the results of the current study support this assumption, suggesting that measurement of HRV during deep sleep are more sensitive to detect ECG abnormalities than those calculated from 24-h recordings.

Regardless of the inconsistencies in the literature, the results of the current study suggest that there is potential to use HRV measures, such as LF/HF ratio, to acutely monitor responses to high-volume endurance exercise. If LF/HF ratio increases post high-volume endurance exercise, this could be used to indicate liability to increased ECG arrhythmia number. Further wearable devices (i.e., phones, fitness tracking watches, etc.) offer a cost-effective way to monitor exercise within 24-h of performance (i.e., whenever they go to sleep) and non-invasively as long as they produce the HRV measure LF/HF ratio during sleep. Future research should continue to assess the relationship between LF/HF ratio and ECG abnormality number after performing high-volume endurance exercise in a large cohort of people, especially those who perform exercise for health benefit. Future studies should endeavor to identify a potential threshold value where any increase in LF/HF ratio above this is of concern.

HRV measures assessed during deep sleep were unchanged as a result of high-volume endurance exercise. Recovery from acute, shorter duration exercise is normally rapid (e.g., Kaikkonen et al., 2007; Michael et al., 2016) and complete recovery might take at least 48 h after high-intensity exercise (Stanley et al., 2013). Our results extend this work and confirm that after 21-days of high-volume endurance exercise participants displayed no differences when measured 72 h after.

### Strengths and Limitations

A strength of this study was that a controlled measurement technique (i.e., 5-lead Holter monitor) was used to quantify outcomes in the study and HRV measures were measured during deep sleep (and over 24-h) rather than averaged over the 24-h monitoring period. There are, however, a number of limitations that should be acknowledged. First, as data were only captured pre- and post-high-volume endurance exercise, application of HRV measures during exercise as a potential predictor of endurance exercise produced ECG abnormality are not known. What is known is that HRV characteristics are substantially reduced during exercise (Michael et al., 2017), so any change in LF/HF ratio might or might not be related to ECG abnormality in this situation and more research is needed. Second, LF/HF ratio is a non-invasive measure that is typically used as an indirect measure of sympathovagal balance (Task Force of the European Society of Cardiology and the North American Society of Pacing and Electrophysiology, 1996), however, this is controversial in the literature (Billman, 2013; Michael et al., 2017). Higher LF/HF ratio post-exercise being a strong predictor of post-exercise ECG arrhythmia number suggest that high-volume endurance exercise was associated with low vagal activation and sympathetic predominance (Eckberg, 1997) leading to increased ECG arrhythmia number. Sympathetic predominance is typically seen in people under chronic life stress (e.g., Lucini et al., 2005) suggesting that high-volume endurance exercise can lead to a similar situation. However, measures of stress were not captured in this study, so it was not possible to assess the influence of this potential confounding variable.

## Conclusion

High-volume endurance exercise is growing in popularity across the lifespan due to perceived health benefits. However, there is a body of evidence in professional athletes suggesting that exercise might have negative CV consequences when the amount of exercise performed greatly exceeds recommendations. Results from the current study in recreationally active people found that baseline ECG arrhythmia number, in particular supraventricular arrhythmia number, was associated with post high-volume endurance exercise ECG arrhythmia number. After controlling for baseline ECG arrhythmia number, the HRV characteristic LF/HF ratio measured during deep sleep was shown to be strongly associated with ECG arrhythmia number after performing high-volume endurance exercise for 21 consecutive days. LF/HF ratio offers promise as a non-invasive measure associated with high-volume endurance exercise-induced ECG abnormality.

## Data Availability Statement

The original contributions presented in the study are available online (doi: 10.26181/1914974) included in Supplementary Material, further inquiries can be directed to the corresponding author.

## Ethics Statement

The studies involving human participants were reviewed and approved by Bendigo Health Care Group Human Research Ethics Committee and La Trobe University Human Ethics Committee (HREC/17/BHCG/9). Written informed consent to participate in this study was provided by the participants’ legal guardian/next of kin.

## Author Contributions

MK, BG, and CL conceptualized the project. DW and KN performed data collection. DW, KN, and SP analyzed the data. DW, MK, and BW statistically analyzed the data. SP, DW, and BG drafted the first draft. DW, MK, CL, and BG adapted the draft to the final manuscript. All authors contributed to the article and approved the submitted version.

## Conflict of Interest

The authors declare that the research was conducted in the absence of any commercial or financial relationships that could be construed as a potential conflict of interest.

## Publisher’s Note

All claims expressed in this article are solely those of the authors and do not necessarily represent those of their affiliated organizations, or those of the publisher, the editors and the reviewers. Any product that may be evaluated in this article, or claim that may be made by its manufacturer, is not guaranteed or endorsed by the publisher.

## Footnotes

1
https://madcow.org.au/event/mad-ride/
